# Supplementary material for: Gestational Weight Gain Relates to DNA Methylation in Umbilical Cord, Which, In Turn, Associates with Offspring Obesity-Related Parameters
Source: Nutrients. 2023 Jul 17;15(14):3175. doi: 10.3390/nu15143175 (PMC10386148; doi:10.3390/nu15143175)
Supplement: Supplementary file 1 [file nutrients-15-03175-s001.zip › Supplemental Table S1.pdf]

Supplemental Table S1. Clinical assessments in the studied subjects included in the screening analysis (genome-wide DNA methylation) and in the validation analysis (pirosequencing).

		Screening	Validation
**Mother**	N	24	87
	Age at conception (years)	30.71 ± 0.9	30.79 ± 0.43
	Pregestational BMI (kg/m^2)	25.27 ± 1.1	24.46 ± 0.45
	1st trimester BMI (kg/m^2)	25.69 ± 1.1	25.13 ± 0.45
	2nd trimester BMI (kg/m^2)	28.39 ± 1.1	27.21 ± 0.43
	3rd trimester BMI (kg/m^2)	30.66 ± 1.1	29.19 ± 0.43
	1st trimester Weight gain (kg)	1.80 ± 2.5	1.61 ± 0.29
	2nd trimester Weight gain (kg)	7.20 ± 2.9	6.10 ± 0.33
	3rd trimester Weight gain (kg)	4.90 ± 2.5	4.94 ± 0.29
	Total GWG (kg)	16.19 ± 0.6	14.31 ± 0.56
	Gestational obesity (%)	65	36.8
**Newborn**	N	24	87
	Gender (%F)	50%	42%
	Gestational age (week)	40.29 ± 0.2	39.79 ± 0.11
	Birth weight (kg)	3.45 ± 0.53	3.29 ± 0.28
	Birth weight-SDS	0.30 ± 0.1	-0.01 ± 0.06
	Birth length (cm)	50.17 ± 0.3	49.49 ± 0.16
	Birth length-SDS	0.07 ± 0.2	-0.22 ± 0.09
**Follow-up at age 6 years**	N		61
	Gender (%F)	-	44
	Age (years)	-	5.81 ± 0.12
	Weight (kg)	-	22.37 ± 0.66
	Weight-SDS	-	-0.01 ± 0.15
	Height (cm)	-	16.87 ± 0.30
	Height-SDS	-	-0.10 ± 0.15
	BMI (kg/m^2)	-	16.87 ± 0.30
	BMI-SDS	-	0.24 ± 0.14
	Fat mass (%)	-	24.00 ± 1.19
	Fat mass-SDS	-	0.31 ± 0.20
	Δ BW-BMI SDS	-	0.26 ± 0.16
	Waist (cm)	-	57.23 ± 0.94
	cIMT (cm)	-	0.037 ± 0.001

Data are expressed as mean ± SEM. BMI: Body-mass index; SDS: Standard-deviation score; GWG: gestational weight gain; Δ BW-BMI: BMI-SDS at 6yr - Birthweight-SDS; cIMT: carotid intima-media thickness.
