# Supplementary material for: Gestational Weight Gain Relates to DNA Methylation in Umbilical Cord, Which, In Turn, Associates with Offspring Obesity-Related Parameters
Source: Nutrients. 2023 Jul 17;15(14):3175. doi: 10.3390/nu15143175 (PMC10386148; doi:10.3390/nu15143175)
Supplement: Supplementary file 1 [file nutrients-15-03175-s001.zip › Supplemental Table S2.pdf]

Supplemental Table S2: Pyrosequencing primers and PCR conditions.

Gene		*Primers*	*Annealing temperature*	*Fragment size (bp)*
**SETD8**	Fw	GAATTTGGGGTTTTTTAATGGTAGT	57ºC	357
	Rv	**BIOT**-ACCTCTAACCATAACTCCACTCTATCA		
	Seq	AGTAGTTGGAGTT		
**TMEM214**	Fw	AAAGGAAAGAAAAATAGAAATAGTTTTATT	54ºC	201
	Rv	**BIOT**-AAACTTATAATCCCTCTCCTAAACC		
	Seq	TGGTTTTTATAT		
**SLIT3**	Fw	GTTTAGTTGTTTGTGGAAAAGTAGG	56ºC	306
	Rv	**BIOT**-ACCCTAATATAATAAAATTACTCCCTTCTA		
	Seq	GAGAGAATTATATAG		
	Fw	TGTGGTTGTTTGTAGTTTTTTGTGTA	57ºC	294
**RPTOR**	Rv	**BIOT**-CAAACTCAACTCCCAAACCTATCT		
	Seq	AGGTTTAAAGTAT		
**HOXC8**	Fw	AGTTGGGGTTGGTTTATTATTTTTT	58ºC	483
	Rv	**BIOT**-AACCCCAAACCAACTAACTCC		
	Seq	TGGATTGATGAA		
