# Supplementary figures and images for: Gestational Weight Gain Relates to DNA Methylation in Umbilical Cord, Which, In Turn, Associates with Offspring Obesity-Related Parameters
Source: Nutrients. 2023 Jul 17;15(14):3175. doi: 10.3390/nu15143175 (PMC10386148; doi:10.3390/nu15143175)

## Slide 1
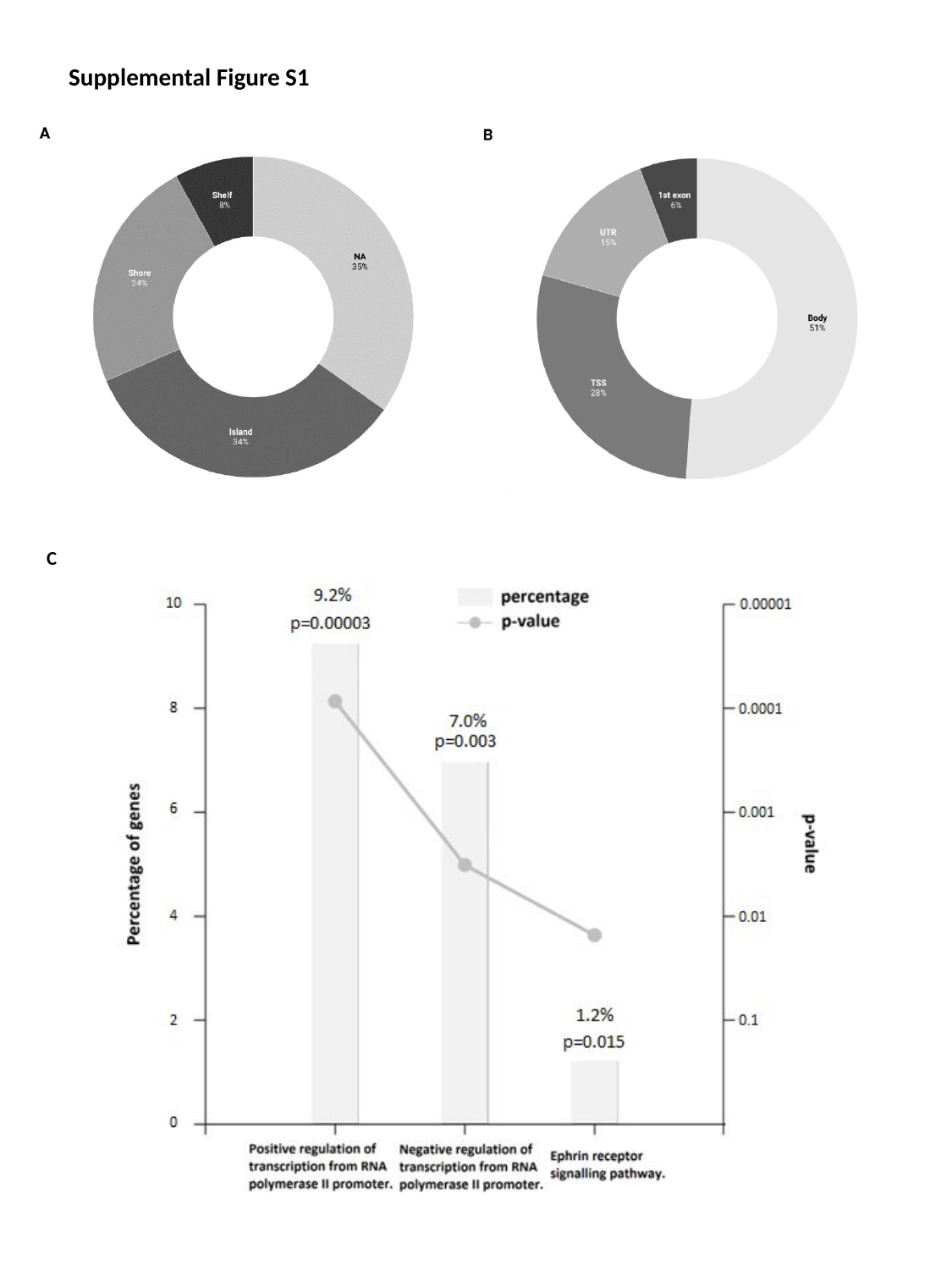

Supplemental Figure S1
C

Supplement: Supplementary file 1 [file nutrients-15-03175-s001.zip › Supplemental Figure S1.pptx]
